# Putting non-communicable disease data to work in Vietnam: an investigation of community health surveillance capacity

**DOI:** 10.1186/s12889-023-14986-4

**Published:** 2023-02-14

**Authors:** Thu Nam T. Nguyen, Thi Tho T. Nguyen, Bao Quoc Tran, Cong Tuan Pham, Kelly E. Perry, Tilahun Haregu, Brian Oldenburg, Paul Kowal

**Affiliations:** 1 FHI 360 Vietnam Office, 17Th Floor, Capital Tower, 109 Tran Hung Dao Street, Hanoi, Vietnam; 2 Noncommunicable Disease Prevention and Control Department, National Institute of Hygiene and Epidemiology, 1 Yec Xanh Street, Hanoi, Vietnam; 3 Noncommunicable Disease Prevention and Control Department, General Department of Preventive Medicine, Ministry of Health, 138 Giang Vo Street, Hanoi, Vietnam; 4 FHI 360, Asia Pacific Regional Office, 19Th Floor, Tower 3 Sindhorn Building 130-132 Wireless Road Kwaeng Lumpini, Khet Phatumwan, Bangkok, Thailand; 5 Baker Heart & Diabetes Institute, 75 Commercial Rd, Melbourne, Melbourne, Australia; 6 International Health Transitions, Canberra, Australia; 7 Research Institute for Health Sciences, Chiang Mai University, 239 Huay Kaew RdMueang Chiang Mai District, Chiang Mai, Tambon Su Thep, Thailand

**Keywords:** Non-communicable diseases, Hypertension, Diabetes, Surveillance, Community health, Vietnam

## Abstract

**Introduction:**

Despite the public health system’s critical role in non-communicable disease (NCD) surveillance in Vietnam, limited evidence exists on the implementation of NCD surveillance activities within these systems and the need for capacity building across different system levels to meet expected NCD surveillance goals. This study aimed to evaluate the status of and describe factors affecting the implementation of NCD surveillance activities and to identify the NCD surveillance capacity building needs of the public health system in Vietnam.

**Methods:**

We used a mixed-methods approach in four provinces, conducting self-completed surveys of staff from six Preventive Medicine Institutes (PHI), 53 Centres for Disease Control (CDC) and 148 commune health stations (CHS), as well as 14 in-depth interviews and 22 focus group discussions at four PHI, four CDC, and eight district health centres and CHS.

**Results:**

Study findings highlighted that although Vietnam has a well-functioning NCD surveillance system, a number of quality issues related to NCD surveillance data were salient. Multifactorial reasons were identified for incomplete, unconfirmed, and inaccurate mortality data and current disease surveillance data. Data on NCD management and treatment were reported to be of better quality than data for screening, targeted treatment, and counselling communication. Main factors affected the effective implementation of NCD surveillance, namely lack of complete and specific guidelines for NCD surveillance, limitations in human resource capacity within NCD departments, and shortage of funding for NCD surveillance activities.

**Conclusion:**

Study findings provide practical strategies for strengthening health system capacity for NCD surveillance through developing policies, guidelines, and standardised tools to guide NCD surveillance and a road map for integrated NCD surveillance, developing training packages and manuals for all levels of the health system, and conducting utilisation-focused surveillance training programs.

**Supplementary Information:**

The online version contains supplementary material available at 10.1186/s12889-023-14986-4.

## Key messages

- What is already known?

Vietnam’s public health system has a critical role in non-communicable disease (NCD) surveillance, yet surveillance structures are not well understood and underdeveloped, with continual reliance on global NCD estimates as NCD data at the national level is lacking. Understanding and bolstering NCD surveillance capacity could strengthen the NCD surveillance system and meet expected NCD surveillance goals.

- What does this study add?

This was the first study to investigate the context and capacity building needs of NCD surveillance in Vietnam which highlighted several quality issues related to NCD surveillance data, including lack of guidelines for NCD surveillance, limitations in human resource capacities within the public health system, and shortage of funding for NCD surveillance activities.

- What do the new findings imply?

A comprehensive understanding of the context and capacity building needs of NCD surveillance can support the development of policies, guidelines, and standardised tools to guide NCD surveillance and training manuals at all levels of Vietnam’s health system.

## Introduction

Monitoring country progress towards the Sustainable Development Goals’ health-related targets, including those on non-communicable diseases (NCD), has resulted in widespread uptake of global health estimates from the World Health Organization (WHO) and the Institute of Health Metrics and Evaluation Global Burden of Disease study [1]. The use of these estimates has been necessary because of a dearth of data for many indicators and the desire for cross-country comparability [2, 3].

However, national health studies or surveillance systems’ estimates can differ from global health estimates. For example, using data from the most recent Global Burden of Disease study, the percentage of total deaths attributed to NCD in Vietnam was 78% in 2014, 79% in 2016, and 80% in 2018 [4]. In contrast, official national estimates of deaths attributed to NCD according to the Vietnam Ministry of Health (MOH) were 73% in 2014, 63% in 2016, and 80% in 2018 [5]. Discrepancies between global and national estimates could be reduced through investments in analytical capacity at sub-national levels, improving coverage and accuracy of capturing health-related data in communities and leveraging data from multiple sources for health-related decision-making [6].

In Vietnam, NCD surveillance relies on both curative and preventive systems. The General Department of Preventive Medicine, the MOH, serves as the central management body providing NCD surveillance technical guidelines and policy development and monitors their implementation nationwide. At the regional level, the six public health institutes (PHI) are responsible for two main functions: the implementation of national surveys on risk factors, nutrition and morbidity as well as training provision to provinces in assigned geographic areas. At the province level, Centers for Disease Control (CDC) oversee the development of local budgets and activity plans for NCD surveillance as part of the general NCD program. They aggregate provincial NCD data from hospitals and preventive units at district and commune levels to then report to the MOH. CDC also conduct NCD surveillance training to a cadre of districts and communes in the provinces. At grassroots levels, commune health stations (CHS) serve as critical nodes in the health information system; CHS collect, synthesise, and manage information and statistics as well as report health data—including data on NCD—to district health centres that aggregate CHS’ health data to report to provincial CDC [7]. The district health centres provide direction and management of all activities by CHS.

Challenges to effective NCD prevention and control in Vietnam include limited awareness of NCD within communities, regional disparities in medical services, healthcare staff shortages, and poor NCD surveillance [7]. Improving staff competency in collecting and using NCD data in CHS can potentially improve NCD prevention and management in Vietnam [8]. Currently, over 11,000 CHS exist–with CHS staff working with more than 100,000 village health workers and community collaborators who play a crucial role in the preventive medicine system [5]. CHS, as the lowest medical unit of the public health care system, are responsible for primary health care, and public health activities such as health communication, education, and disease outbreak detection and reporting [7].

In 2015, the Vietnam government endorsed a national Strategy for NCD Prevention and Control. While Vietnam has made steady progress on different facets of NCD surveillance, a stronger and more integrated NCD data collection system needs to be established along with a trained workforce able to analyse and use data at CHS and community levels [9]. Current literature presents empirical data on the capacity of system-specific NCD surveillance packages and diseases, but not the whole NCD surveillance system capacity [10]. Vietnam would benefit from a review of the NCD surveillance system organisation, function, and capacity at each level of the current health system to identify how to improve quality and maximise the efficacy of its NCD surveillance system to align with the country’s NCD roadmap.

This study assessed the implementation status of NCD surveillance activities in Vietnam, described factors affecting the implementation of NCD surveillance activities and identified capacity building needs of the public health system for NCD surveillance.

## Methods

### Study design and period

A cross-sectional, mixed-method study was conducted between July and December 2020 to define the current state of NCD data capture within existing systems and to assess NCD data collection, reporting, and analytical capacity needs at CHS and preventive medicine systems at provincial, regional, and national levels.

**Fig. 1:**
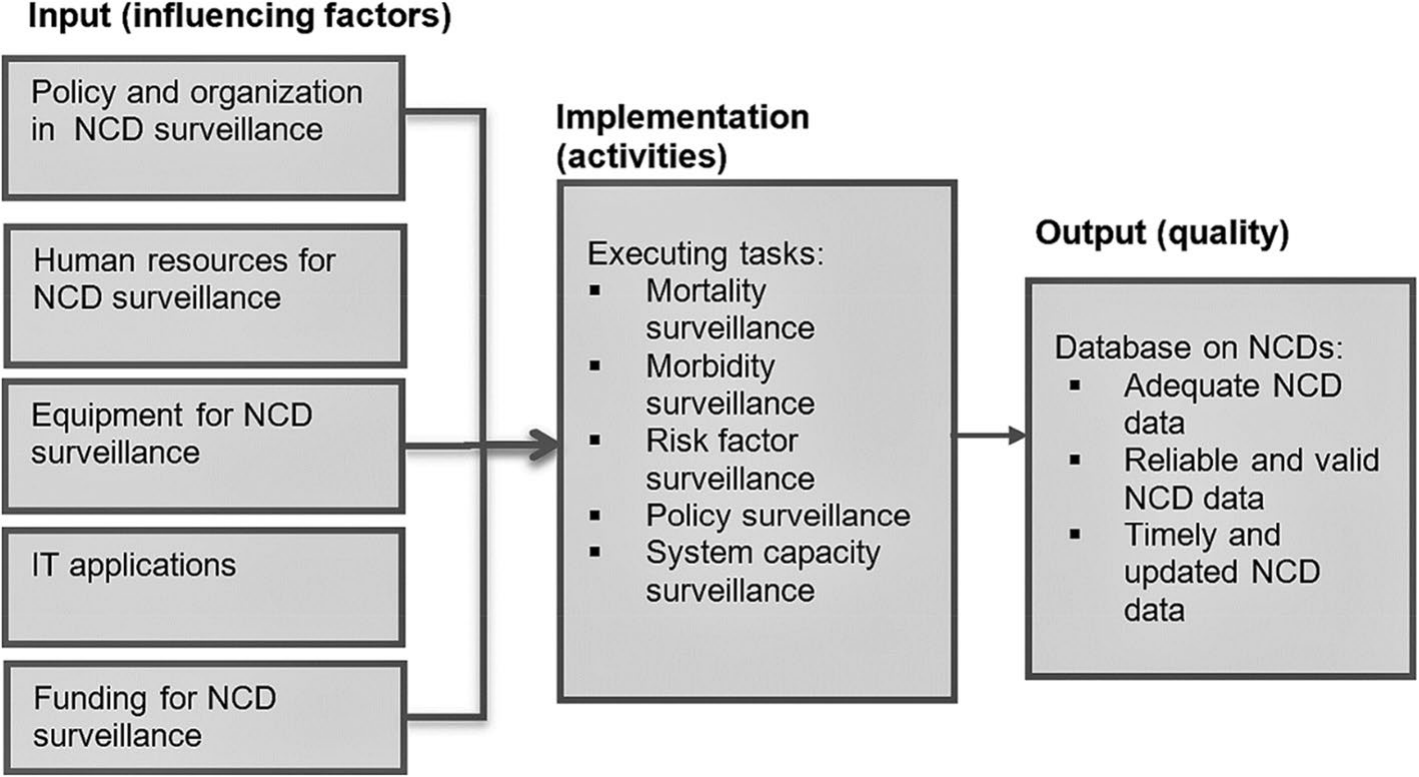
Situational analysis framework adopted from the World Health Organization Health Systems Framework (***)

The analysis was grounded in a theoretical framework that considered input capacity, process and implementation capacity, and outputs, as demonstrated in Fig. 1 [11, 12]. Evaluation of analysis results, was further framed by a conceptual framework based on the WHO’s six health systems building blocks [13, 14].

### Study population

All six Public Health Institutes (PHI) (100%)—the National Institute for Hygiene and Epidemiology, the Pasteur Institute in Nha Trang, the Pasteur Institute in Ho Chi Minh City, the Tay Nguyen Institute of Hygiene and Epidemiology, the Institute of Public Health in Ho Chi Minh city, and the National Institute of Nutrition—and 53 provincial CDC among 63 CDC in Vietnam (84.1%) returned filled questionnaires. Four provinces of the North (Bac Giang), Central (Khanh Hoa), Central highlands (Daklak) and South (Ho Chi Minh City) were selected for primary health care data collection to represent the geographic regions of Vietnam. Four districts, one district in each selected province, were randomly selected where all CHS in the four districts were invited to complete the questionnaire and two CHS were randomly engaged in qualitative inquiries (Table 1).

**Table 1:** Participant type, type of data collection method and number of participants

No	Subjects	Self-administered questionnaire	In-Depth Interview	Focus Group Discussion
	**Public Health Institutes (PHI)**			
1	PHI leaders	1 × 6 PHI = 6 questionnaires	1 × 6 PHI = 6 persons	
2	Faculty leaders and staff of NCD unit			1 × 6 PHI = 6 groups
	**Provincial CDC**			
1	Provincial CDC leaders	1 × 53 CDC = 53 questionnaires	1 × 4 CDC = 4 persons	
2	Staff of NCD Unit			1 × 4 CDC = 4 groups
	**District Health Centres**			
3	Leaders of the Centres		1 × 4 centres = 4 persons	
4	Head and staff of NCD unit			1 × 4 centres = 4 groups
	**Commune Health Stations (CHS)**			
5	Head and staff of CHS	1 × 148 CHS = 148 questionnaires		1 × 8 CHS = 8 groups
	**Total**	**207**	**14**	**22**

The study team also reviewed the following materials relevant to NCD surveillance: policy documents related to and existing documents and guidelines on national and regional NCD surveillance; technical guidelines on NCD surveillance issued to CHS; NCD surveillance plans (NCD surveillance components within NCD control plans, capacity development and implementation, and finance); reports related to NCD control and monitoring at CHS; and existing logbooks, tools, and reporting templates on NCD data at all levels.

### Data collection

The study used a self-administered survey to collect data on capacity, utilisation, and gaps within the collection, management, and use of NCD information at the PHI, CDC, and CHS. The questionnaire was developed by the research team based on the functions assigned to each level regarding NCD surveillance. The questions were divided into three parts: implementation of NCD surveillance at each of the levels (PHI, CDC, and CHS) through multi/single selection options and open-ended questions; the capacity and influencing factors through table/matrix filling, multi selection options and open-ended questions; and the obstacles, solutions and needs with additional types of ratings (Supplementary Table 3). In-depth interviews and focus group discussions contained questions on the NCD surveillance system’s major components, challenges faced during implementation, and training and capacity-building needs of organisations. The set of questionnaires and guidelines for in-depth interviews and focus group discussions were reviewed by a group of NCD experts from all levels of the NCD surveillance system before submitting to the Institutional Review Board for review. The questionnaires were distributed to CDC and collected via mail. Questionnaires for PHI and CHS were distributed and collected by the author team in data collection field trips.

All in-depth interviews and focus group discussions were conducted in the PHI, CDC, and CHS. Respondents were asked about human resources, facilities, equipment, and software (information technology) currently being used for NCD surveillance. Each in-depth interview and focus group discussion lasted approximately 45 and 60 min, respectively (see Supplementary Table 1 for detailed data collection tools and methods).

### Data analysis

Quantitative data were managed and cleaned using EpiData 3.1 [15]. Descriptive analyses (frequency distribution) were summarised using SPSS version 19.0 [16]. The quality assessment of collected NCD data, including data completeness and accuracy, was self-rated by interviewees against NCD data templates assigned to them.

Regarding qualitative data, in-depth interviews and focus group discussions were audio-recorded, transcribed, and saved as Microsoft Word files. Personal identifiers were deleted from transcription files. The first, second, third, and fourth authors (N.T.T.N., N.T.T.T., T.Q.B., and P.C.T.) conducted thematic analysis on qualitative data using a pre-existing coding scheme as well as emerging themes. In addition, the study team reviewed technical guidelines on CHS NCD surveillance, reports related to NCD control and monitoring at CHS, and existing logbooks, tools, and reporting templates on NCD data at CHS. While data were collected at the regional level, the situational analysis primarily focused on the country-level context and did not provide an in-depth examination of regional differences in NCD surveillance capacity.

## Results

### Policy review of the NCD surveillance system and data in Vietnam

In 2019, the Ministry of Health issued a new circular on health statistics indicators (20/2019/TT-BYT), which included increased indicators on NCD, mandating these to the provincial, district, and commune health care services and management systems, as demonstrated in Table 2. Population-based data and indicators are reported from CHS to district and provincial management systems, and finally to the Ministry of Health, with primary indicators regarding mortality and morbidity. NCD treatment and care data from hospitals are reported directly to provincial departments of health and the Ministry of Health. Morbidity data could be duplicated if individuals access services from both hospitals and CHS. The unidirectional reporting flow lacks technical guidelines on NCD surveillance system structure and function, causing frequent misunderstandings about NCD surveillance, hence each level of the health management system has utilised and leveraged its own data to inform planning.

**Table 2:** Selected health statistics indicators on NCD and reporting flow^a^

Indicators	Indicator collection method	Ministry of Health	Public Health Institutes	Provincial Health Department/CDC	District Health Centre	Commune Health Stations	Hospitals
**Risk factor**							
Tobacco use, alcohol consumption, etc	Surveys (WHO, GSHS, GATS, national survey)^b^	Collect and analyse information	Technical support				
**Impact factor**							
Mortality and causes	Periodical report Surveys	Collection of information from facilities under MOH, and analyse from all sources	Technical support	Collection and report information from facilities under province and from district to MOH	Collect and report information to the province	Collect and report information to the district	Collect and report information to province or Ministry of Health
Malnutrition, obesity	Surveys	Collect and analyse information	Collect and analyse information				
**NCD**							
Diagnosis and treatment of hypertension, diabetes, COPD, asthma, mental disorders	Periodic reports National Surveys	Collection of information from facilities under MOH, and analyse from all sources		Annually collection and report information from facilities under province and from district to MOH	Quarterly collect and report information to the province	Quarterly collect and report information to the district	Collect and report information to province/ MOH
**System Capacity**							
CHS NCD prevention and treatment	Periodical report Surveys (SARA)^b^	Collect and analyse information		Collection and report information to MOH			

^a^ selected from Circular 20/2019/TT-BYT, issued by Health Minister on July 2019

^b^ *WHO* World Health Organization STEPwise Approach to NCD Risk Factor Surveillance, *GSHS* Global School-based Student Health Survey, *GATS* Global Adult Tobacco Survey, *SARA* Service Availability and Readiness Assessment

PHI are not involved in the health data reporting system. Rather, these institutes provide information to the Ministry of Health and stakeholders on health system capacity and NCD risk collated through periodic standardised surveys such as WHO’s risk factor surveillance survey, Global School-based Student Health Survey, Vietnam General Nutrition Survey, and others. PHI, including the Institutes of Hygiene and Epidemiology and Pasteur Institute, as well as CDC, are responsible for synthesising and reporting this information and have been involved in mortality surveillance from the beginning of 2020. The National Institute of Nutrition and Institute of Public Health Ho Chi Minh City are not involved with mortality surveillance, as these institutes are not mandated to collect and monitor mortality data and have not been assigned this task by the Ministry of Health.

As demonstrated in Table 3, all CDC and CHS have been assigned to collect routine NCD data. Only PHIs and CDC were involved in risk factor surveillance, primarily with the WHO risk factor surveillance surveys, and in system capacity surveillance. CHS have not yet engaged in risk factors and system capacity surveillance.

**Table 3:** Number (%) of preventive medicine organisations implementing NCD surveillance components

Implementation of surveillance components	PHI (*n* = 6)	Centre for Disease Control (CDC) (*n* = 53)	CHS (*n* = 148)
Mortality surveillance	4 (66.7%)	53 (100%)	146 (98.6%)
Collecting data from commune level	4 (66.7%)	53 (100%)	126 (85.1%)
Conducting mortality surveys^a^	0	1 (1.9%)	20 (13.5%)
Morbidity surveillance	6 (100%)	53 (100%)	143 (96.6%)
Hypertension/CVS	6 (100%)	53 (100%)	143 (96.6%)
Diabetes	6 (100%)	50 (94.3%)	120 (81.1%)
Chronic obstructive pulmonary disease (COPD)/ Asthma	3 (50.0%)	31 (58.5%)	87 (58.8%)
Risk factor surveillance^a^	6 (100%)	53 (100%)	-
WHO risk factor survey	6 (100%)	53 (100%)	-
Tobacco survey	1 (16.7%)	15 (28.3%)	-
School health survey	0	1 (1.9%)	-
Policy/system capacity surveillance^a^	**2 (33.3%)**	**32 (60.4%)**	**-**
Collecting data from grassroot level	2 (33.3%)	30 (56.6%)	-
Conducting surveys	1 (16.7%)	14 (26.4%)	-

^a^periodic sample surveys

### Surveillance data quality

All PHI and approximately one-third of the CDC have established NCD databases, though they consist primarily of screening, treatment, and mortality data. Study findings indicate that CHS were likely to perceive their data quality was sufficient compared to PHI and CDC, especially regarding data completeness and accuracy (see analysis of the self-assessment on data quality, timeliness, and completeness in Supplementary Fig. 1).

All PHI, CDC, and most CHS implemented morbidity surveillance, focusing on hypertension and type 2 diabetes. A limited number of these units were involved in chronic obstructive pulmonary disease (COPD) and asthma diagnosis and management surveillance even though such surveillance was required in CHS. NCD morbidity data were collated from the NCD management book and other medical examination books at CHS, as noted as follows:

“The morbidity data is collected through reports from the commune level and statistics from the medical examination book. There is some data from the screening and integrated examination [for older adults], reporting from examinations in the community.” (In-depth interview with CDC leader)

Data inaccuracy and incompleteness occurred often, including underreporting of new NCD cases and erroneous or incomplete NCD records from CHS. Qualitative data results from this study indicated that many CHS staff only obtained training on how to record, compile, and report data, but not how to collect data and check sources. The following quote illustrates data incompleteness:

“This data [NCD treatment] report is not complete because many clinics and hospitals are treating this kind of disease and we cannot collect their data. Some people are treated in other places. (District Health Centre group discussion)

Regarding the quality of mortality data, multiple sources were used to determine the number of deaths recorded by CHS, ranging from village health workers (70.9% of surveyed CHS relied on this source, *n* = 148), population collaborators (the system established by the government to collect vital information on birth and death and support family planning implementation) (70.9%), and judicial and commune public security officials (42.6%) (See Supplementary Table 5).

Nearly 86% out of 148 CHS reported that they have a system for determining cause of death, drawing from multiple sources, with 54.1% collating information from interviews with the deceased individuals’ relatives, 49.3% based on death certificates of which cause of death was reported by family members of the deceased, and 37% based on hospital discharge papers prior to death. The least used source of mortality data was the mortality survey conducted with the deceased individuals’ families (13.5%). A CHS representative elaborated:

“I ask the village health workers for the cause of death. If it is a case referred from higher (healthcare) levels, I obtain the cause of death information from hospitals and record it in the A6 logbook [this is the mandated documentation for all deaths]. In addition… information is also obtained from the village population collaborator or relatives. We [the CHS] have not re-examined the diagnosis of the cause of death, but only reviewed the list.” (CHS group discussion)

Findings from secondary data analysis show that approximately 70% of CHS staff shared that their station reported cause of death with ICD-10 codes according to the new Circular on health statistics. “Old age” was frequently observed as the cause of death for individuals 60 years and older. Instead of reporting the cause of death details with ICD-10 codes listed in the Circular, some surveyed stations reported grouped causes, such as “cancer” (e.g., C00-C79), even though 51.3% (*n* = 148) were trained in collecting information on mortality and 46.6% (*n* = 148) trained in diagnosing and coding mortality causes. In addition, no CHS reported having guidelines and standard tools that supported collecting data for cause of death identification.

Qualitative interview and focus group results demonstrated that provincial PHI and CDC staff collated mortality data according to the statistical system reporting data (A6 logbook) from CHS without any inspection and supervision on the quality of these metrics:

“The PHI only summarise the reporting results sent from lower levels and cannot check the accuracy of the data…There is no available mortality data from hospitals” (Group discussion with PHI staff)

### Factors affecting the implementation of NCD surveillance activities and capacity building needs

**Fig. 2:**
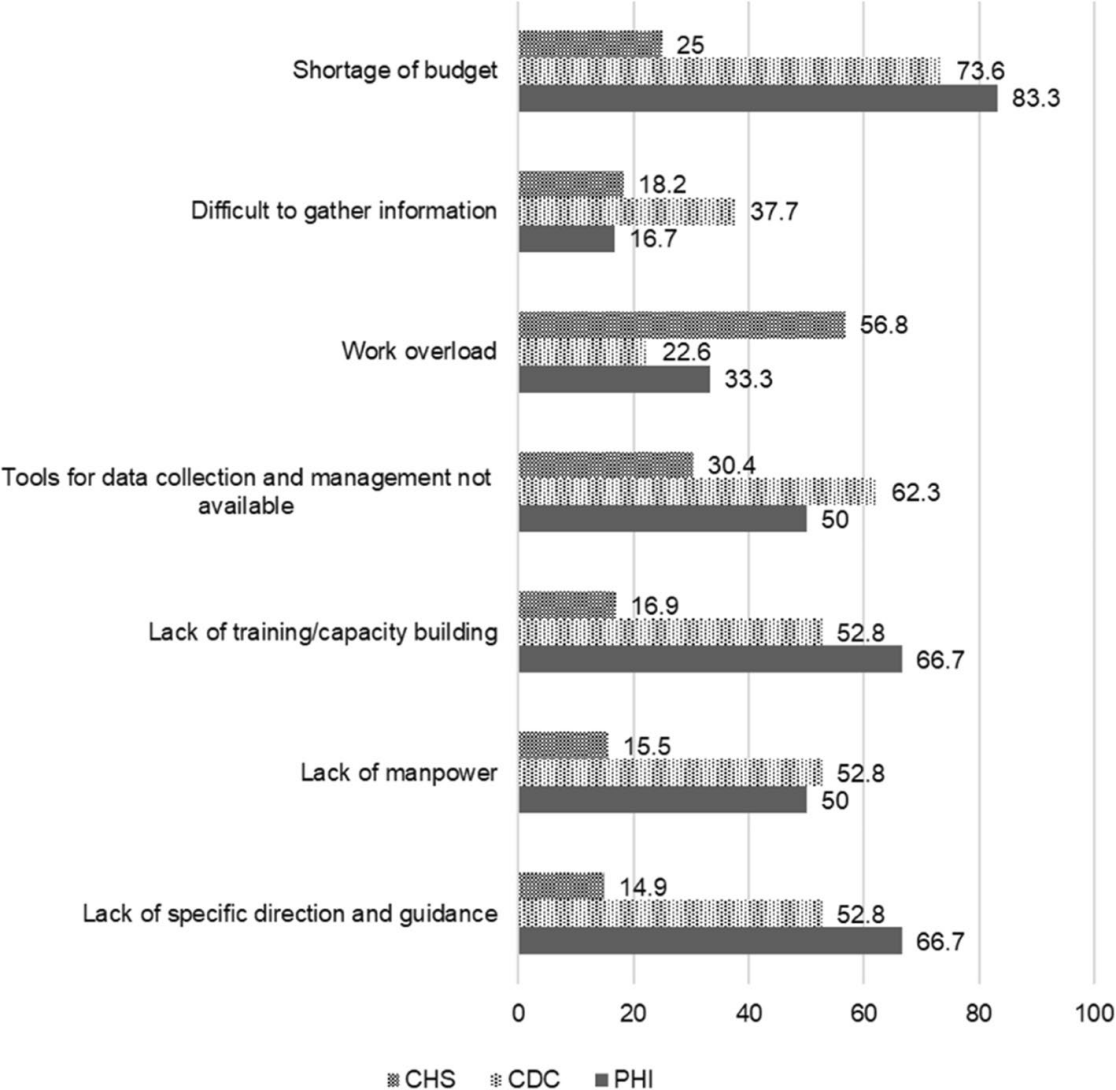
Perception of challenges for NCD data collection and report at all levels

Survey results (Fig. 2) demonstrated similarities in the perception of challenges that PHI and CDC face, including budget shortages (83.3%, *n* = 6 and 73.6%, *n* = 53, respectively), lack of staff training and capacity building (66.7 and 52.8%), and lack of specific guidelines (66.7 and 52.8%). High workloads were identified as a primary challenge for CHS (56.8%). The following subsections provide details regarding capacity issues.

#### Policy and governance

The NCD framework and surveillance indicators have been established, with NCD policy documents on control and surveillance gradually being refined. However, a lack of national regulation and technical guidelines on NCD surveillance system structure and operations persists. In addition, guidelines for statistics and reporting (including NCD reporting) provide no specific guidance on coding standards or definitions for disease descriptions, particularly for morbidity data, though instructions on recording cause of death with ICD-10 codes for reporting mortality data do exist. Several CHS still use outdated regulations, impacting NCD data content and quality. Technical advisory agencies, such as the WHO, have provided resources to develop and standardise materials for NCD risk and health system capacity surveys. In addition to technical tool availability, such support assists in establishing a surveillance system structure and governance for periodical survey implementation.

#### Human resources

Among surveyed CHS, more than 80% (*n* = 148) have ever participated in training courses on collecting and managing data related to health education, screening, treatment management, and drugs and equipment for NCD prevention and control. Most CHS (approximately 90%) have assigned staff responsible for NCD who collect information and indicators on NCD according to the national health statistics mandate. However, frequent staff attrition and turnover in stations affect their capacity to collect and report data.

PHI and CDC recognised that they have limited human resources devoted to NCD surveillance, with insufficient numbers of trained staff as noted as follows:

“[We have] new staff without experience who have not been trained about NCD.” (Group discussion with PHI staff)

“We lack human resources for CHS and the CDC. These are young staff members, and they are still rotating a lot.” (In-depth interview of a PHI leader)

The quality of technical support including training and supervision from PHI and CDC to lower-level agencies in NCD surveillance might be an issue when they still lack qualified staff for NCD surveillance. Most PHI developed training materials and guidance on NCD data collection and reporting, though they have not been standardised across the different institutes. Training on statistical reporting has been integrated into treatment management courses.

#### Equipment and tools

All CHS have adequate guidelines and tools, such as logbooks and reporting templates for health statistics data collection and reporting as required, even though they lack guidelines on NCD surveillance. The use of logbooks for health education and hypertension are more common compared to other health topics, with logbook use for COPD and asthma the least common, explained by limited treatment and follow-up for these diseases in the CHS. Because CHS are the focal point for gathering information at the community level, stations manage multiple health programs and projects. However, reporting templates and information systems are not integrated, burdening staff with multiple rounds of data reporting and contributing to overwork in stations.

As presented in Supplementary Table 4, all PHI and 56.6% of CDC have NCD risk factor surveillance guidance documents. However, the availability of tools for morbidity and mortality surveillance was limited among PHI and CDC (33.3 and 16.9% have these tools, respectively). No PHI nor CDC have guidelines and tools for surveillance on health system capacity, morbidity, and NCD. In PHI and CDC, NCD surveillance technical guidelines and tools are not standardised or systematic, with some units equipped with tools if they administer certain surveys. Self-assessment also revealed that all PHI have adequate office equipment for statistics, reporting, and equipment to administer and collate data on NCD surveys. Approximately 87% of CDC and all CHS have adequate essential office equipment.

#### Information technology applications

CHS employed a variety of mechanisms to track and collect data, such as simultaneous use of paper and electronic forms (i.e., Microsoft Excel sheets), NCD management logbooks, outpatient paper-based and electronic records, and different statistical reporting software. Although health information software has been installed in all CHS, the lack of a standardised platform that can streamline information systems for all data management and reporting components has resulted in delays in reporting and inaccurate data. Among surveyed CHS, 52% (*n* = 148) used at least one software program to manage NCD-related data, and 8% (*n* = 148) used two or more types. In 2019, the Ministry of Health released health statistics software based on the District Health Information Software 2 (DHIS2) system. However, the software focused on electronic statistics reporting, not individual data entry and management. Meanwhile, the other local software to collect individual data on examination, treatment and monitoring by different vendors at CHS and other facilities cannot communicate and export data to the health statistics software. The use of local software has also been unstable sometimes. Therefore, all CHS kept paper-based logbooks to record NCD data. One CHS staff member shared:

“Currently we have new software. It’s the only software used in our CHS. I heard that it’s very comprehensive, but I have not explored it thoroughly…. Meanwhile, we still enter data into 12 paper-based logbooks. Paper-based logbooks are our tangible properties which we can retrieve data anytime without fear of losing them someday. We have experienced software changes in our CHS, and we don’t know how to get our data back.” (A group discussion with CHS)

Other information technology application challenges at both CHS and CDC were reported, particularly the lack of an integrated software management system. For instance, software used to report patient examination data (for social health insurance reimbursement purposes) only had examination and treatment information fields. In contrast, other NCD management software at the community level had more fields available. Each software served a different purpose, elaborated on as follows:

“The medical examination and treatment software could not export data by the patient, only containing the number of patient visits. In addition, CDC data reporting personnel have not been trained in medical examination and treatment software.” (A group discussion with CDC staff)

“At present, there is no link between software. Data from the examined patient needs to be entered into a second or third software, and staff [at CHS] are afraid of [using and relying on multiple software].” (A group discussion with CDC staff)

At CDC, the majority (94.3%) used Excel tools and 60.3% used Google Drive tools for statistics and reporting (Supplementary Table 4). However, it is popular (52.8%) to use paper-based reports, as many districts and communes still use this method.

#### Funding for operations

A dearth of NCD surveillance regulatory framework contributed to a lack of funds for routine NCD data collection and statistical analysis. The national targeted population health program, which provides state budgets to socio-economic programs and oversees NCD funds, will terminate at the end of 2021. Nearly all PHI and CDC as well as 81.7% of CHS established plans for NCD surveillance (either separately or integrated with their NCD control plans). However, only 83% (*n* = 6) of PHIs, 86.9% (*n* = 53) of CDC, and 26.4% (*n* = 148) of CHS have funding for NCD surveillance activities (see detailed analysis of the planning and funding for NCD surveillance activities in Supplementary Table 2).

Funding for NCD surveillance within PHI was mobilised from their own budgets (33.3%) and from the targeted population health program budget (66.7%). Similarly, half of the CDC have funding from targeted programs. About 73.5% of CDC have mobilised local funds for NCD surveillance. From 2021 onward, no central government funding will be allocated to targeted programs in the health sector. The budget for provincial agencies is being reviewed and funded by the local government, of which the level of funding is consistent across provinces.

NCD data collection and reporting are integrated as a minor component in the annual plan for CHS. PHI staff shared thoughts as follows:

“Population health targeted program funding has gradually decreased; there are many problems with financial resources as the Institute tends to be self-reliant. The appropriate program was applied but not enough [funding]. There are no resources for scientific research, and implementation of NCD surveillance activities only consists of training and supportive supervision.” (A group discussion with PHI staff)

Most stations did not have a separate budget for data collection and routine reporting, as these activities were considered regular responsibilities within the stations. The budget, if any, will be allocated for other activities and professional tasks.

## Discussion

This was the first study to investigate the context and capacity building needs of the NCD surveillance system in Vietnam, examining the status and factors affecting the current implementation of NCD surveillance activities and identifying capacity building needs of the public health system for implementing NCD surveillance. Analysis of NCD surveillance-related issues has rarely been conducted in low- and middle-income country settings and could contribute to a holistic approach to maintaining high-quality NCD surveillance activities in Vietnam.

The public health system has implemented NCD surveillance activities but not synchronously and comprehensively. In addition, several quality issues related to NCD surveillance data were uncovered in this study. Regarding mortality surveillance, the mortality data were relatively complete, but several limitations in data accuracy (particularly cause of death) existed. Limited activities have been established to supervise nor provide quality assurance of mortality data collected from CHS. In addition, although according to Vietnamese studies [17–19] more than 70% of deaths occurred at home, the majority of CHS did not have standardised tools to collect information from family interviews and did not receive sufficient training on the cause of death identification using these tools. Furthermore, the judicial sector records information reported by families and does not use a standardised tool, contributing to the poor cause of death data quality. Evidence from studies conducted in rural South Africa and Bangladesh demonstrated that verbal autopsy data significantly increased the recording of NCD mortality [20, 21]. In Vietnam, the verbal autopsy has been applied in a number of surveys to identify the cause of death for a better understanding of the morbidity patterns [19, 22–24]. However verbal autopsy application at a national scale has not been studied given the different context of routine data collection and reporting on the cause of death by CHS staff with limited capacity and well setup survey with trained researchers. Considering the evolving technology application to verbal autopsy tools such as using AI for the cause of death diagnosis [25, 26]. It will be useful for Vietnam to conduct a feasibility study on standardised verbal autopsy forms supported by technologies in the context of routine mortality surveillance to provide evidence base recommendation for the application of this method.

Regarding the perceived quality of surveillance data, most units at PHI and CDC claimed that data reporting was often conducted in a timely manner. However, these units believed that data quality was considered average or below average and that it would be difficult to sufficiently assess data for completeness and accuracy. CHS perceived a higher level of data accuracy than PHI and CDC. A high workload was considered a prominent challenge for NCD data collection and reporting compared to technical issues such as lack of capacity and guidance. Meanwhile, PHIs and CDC that provided training and guidance to CHS perceived a lower level of data accuracy and attributed this to a lack of technical guidance. The lack of technical guidelines on NCD surveillance, standardised process of data collection and quality control, and manpower from PHIs and CDCs for monitoring possibly contributed to poorer perceptions of data quality at CHS.

It is recommended to consolidate the organisational structure of the NCD department within PHI and CDC to have adequate human resources and assign a dedicated team to manage NCD surveillance. In addition, only approximately half of CHS have received training to use data recording books and statistical reporting. A study conducted in Vietnam highlighted similar capacity building needs of the public health system staff for the prevention and control of NCD in CHS [27]. Similarly, Vietnam's primary health care system has been reported to have limited NCD service capacity [9]. A qualitative study in urban Hanoi reported that the NCD prevention and control workforce at CHS would be strengthened by having more health workers with NCD-specific training and skills [28].

The qualitative data indicated that staff were aware of underreported cases of NCD when people access health facilities outside the residential location. Despite data source collection at the provincial level, cases were likely inaccurate because reporting data sources were aggregated, not patient level. Although some CHS and hospitals have used the software to collect individual data on care and treatment, no data connectivity between the care and treatment data software and DHIS2 based health statistics software cause an increased likelihood of data error, which is also reported elsewhere [29]. In addition, although some CHS and hospitals could export patient-level data from their health information system (HIS), the issue still exists due to the lack of a master patient index within the health care system in Vietnam currently. Therefore, factors related to the use of information technology also play an important role in the implementation and quality of the surveillance system in Vietnam. No integrated software for NCD statistics, reporting, and surveillance exists. Most PHI and CDC use Excel tools and a cloud-based drive for statistics and reporting. Despite the presence of different software used for health programs and statistics, health information is fragmented due to a lack of data interoperability, contributing to a high workload related to NCD data management and statistics at CHS. Improving the digital health information system by developing and implementing a plan for digital transformation towards an integrated NCD surveillance database could improve the availability and quality of data and ease workloads at CHS.

This study identified a complex set of interrelated health system factors impacting the implementation of NCD surveillance in Vietnam and related capacity building needs. Concerning policy and governance, although the policy environment for NCD surveillance has improved and an NCD framework and indicators have been established, no complete and separate guideline for NCD surveillance exists. Higher-level guidance on statistical reporting focused on meeting the requirements of individual programmes and projects. Many CHS have not yet been instructed to implement statistical reporting according to newly issued regulations. A similar finding was reported in a qualitative study in Hanoi which demonstrated that health staff at CHS noted the lack of NCD informational materials for management and planning [28].

As suggested by our study findings, the national NCD program in Vietnam should review policies, legal documents, and technical guidelines regarding NCD surveillance and develop new, amended, and supplemented legal documents for the collection, management, reporting, and use of NCD information at all levels of the health sector. The program could then develop a simple and standardised NCD reporting form that captures required NCD indicators to meet national data needs and minimise overlap of reporting responsibilities. In addition, standardised NCD reporting structures could be developed to use results at primary healthcare levels for patient care.

Funding has a large impact on the sustainability of the NCD surveillance system. No direct funding stream for NCD data collection, management, and reporting current exists, as NCD surveillance funding has only been integrated into specific programs such as NCD screening and care communication campaigns [9]. Lack of direct funding might affect the quality of NCD data collection and reporting because NCD data have not been collected routinely and the scope of data collection has been modified over time [28]. To address this, it is recommended to strengthen policy advocacy activities at primary healthcare levels by regularly disseminating NCD information to the government and other stakeholders to receive feedback and mobilise communities, businesses, and social organisations to prioritise NCD prevention [30]. A qualitative study also underscored the importance of facilitating stronger government commitments to NCD prevention and control, capacity building, professional recognition, and provision of incentives based on available resources [31].

Our study aimed to understand NCD surveillance at all levels of the health system, including technical support from regional PHI, technical management and direction from CDC, and implementation at primary healthcare levels (district and CHS). All surveyed CHS are in or adjacent to provinces where PHI are located and are likely to receive more technical support and monitoring compared to CHS located in provinces without such PHI. In addition, information bias could have occurred, as the study’s quantitative data on reporting accuracy was self-reported. To mitigate such bias, researchers explored NCD data and indicators collected from CHS in detail. Logbooks used at CHS were also reviewed during qualitative data collection visits.

## Conclusion

Study results indicate that although Vietnam has a NCD surveillance system integrated into health system management from community to central levels, many data quality issues persist. To improve NCD surveillance quality in Vietnam, the Ministry of Health should develop policies, guidelines, and standardised tools to guide data collection systems for integrated NCD surveillance, develop training packages and manuals, and conduct utilisation-focused training programs using face-to-face and online training. Additionally, the Ministry of Health should strengthen policy advocacy activities for funding for NCD surveillance activities by regularly disseminating NCD information to the government and other stakeholders and mobilising communities, businesses, and social organisations to prioritise NCD prevention.

## Supplementary Information

**Additional file 1:** **Supplementary Table 1. **Data collection tools and methods. **Supplementary Table 2. **Planning and funding for NCD surveillance activities. **Supplementary Table 3. **Questionnaire sections for the self-administered survey. **Supplementary Table 4. **The specialized documents for NCD surveillance. **Supplementary Table 5. **Source of information for identifying the number of death and cause of death in CHSs. **Supplementary Figure 1.** Self-assessment of data quality, timeliness, and completeness.

## Data Availability

The data that support the findings of this study are available from the United Kingdom’s (UK) Foreign and Commonwealth Development Office Prosperity Programming—Better Health Programme but restrictions apply to the availability of these data, which were used under license for the current study, and so are not publicly available. Data are however available from the authors upon reasonable request and with permission of the United Kingdom’s (UK) Foreign and Commonwealth Development Office Prosperity Programming—Better Health Programme.

## References

[1] Lozano R, Fullman N, Abate D, Abay SM, Abbafati C, Abbasi N, Abbastabar H, Abd-Allah F, Abdela J, Abdelalim A (2018). Measuring progress from 1990 to 2017 and projecting attainment to 2030 of the health-related sustainable development goals for 195 countries and territories: a systematic analysis for the global burden of disease study 2017. The Lancet.

[2] Murray CJL, Frenk J, Piot P, Mundel T (2013). GBD 2.0: a continuously updated global resource. Lancet.

[3] Boerma T, Victora C, Abouzahr C (2018). Monitoring country progress and achievements by making global predictions: is the tail wagging the dog?. The Lancet.

[4] Global Health Data Exchange GBD Results Tool [http://ghdx.healthdata.org/gbd-results-tool].

[5] Ministry of Health. Health statistics yearbook - 2020. In. Hanoi: Vietnam Medical Publishing House; 2021:225.

[6] Kostova D, Richter P, Van Vliet G, Mahar M, Moolenaar RL (2021). The role of noncommunicable diseases in the pursuit of global health security. Health Secur.

[7] Hattori K, Uda H, Hitomi Y, Yano R, Saijo T, Watanabe N, Satomi M, Yoshida A, Oishi O, Yamashita T (2018). The current situation and agendas in the prevention and control of non-communicable diseases in Vietnam. Nihon Koshu Eisei Zasshi.

[8] Tran TA, Hoang VM, Adler AJ, Shellaby JT, Bui VT, McGuire H, Le TTH, Nguyen TV, Hoang TA, Le MD et al. Strengthening local health systems for hypertension prevention and control: the Communities for Healthy Hearts program in Ho Chi Minh City, Vietnam. J Glob Health Sci. 2020;2(1):1–19.

[9] Duong DB, Minh HV, Ngo LH, Ellner AL (2019). Readiness, availability and utilization of rural vietnamese health facilities for community based primary care of non-communicable diseases: a crosssectional survey of 3 provinces in Northern Vietnam. Int J Health Policy Manag.

[10] Kroll M, Phalkey RK, Kraas F (2015). Challenges to the surveillance of non-communicable diseases–a review of selected approaches. J BMC public health.

[11] Charmaz K (2014). Constructing grounded theory.

[12] Charmaz K (2006). Constructing grounded theory: A practical guide through qualitative analysis.

[13] World Health Organization. Monitoring the building blocks of health systems: a handbook of indicators and their measurement strategies. In. Geneva: WHO Press, World Health Organization; 2010.

[14] World Health Organization. Everybody's business -- strengthening health systems to improve health outcomes : WHO's framework for action. In. Geneva: WHO Press, World Health Organization; 2007.

[15] Christiansen TB, Lauritsen JM (2010). EpiData Software. EpiData - Comprehensive Data Management and Basic Statistical Analysis System.

[16] IBM Corp. IBM SPSS Statistics for Windows. In: IBM SPSS Statistics. vol. 2021, Version 19.0 edn. Armonk: IBM Corp; 2021.

[17] A Global Burden of Disease Data Plus Model to Inform Domestic Decision-Making: In Search of Super-local Data [https://www.cgdev.org/blog/global-burden-disease-data-plus-model-inform-domestic-decision-making-search-super-local-data].

[18] Collaborators GRF (2018). A systematic analysis for the global burden of disease study 2017. J The Lancet.

[19] Hoa N, Rao C, Hoy D, Hinh N, Chuc N, Ngo D (2012). Mortality measures from sample-based surveillance: evidence of the epidemiological transition in Viet Nam. Bull World Health Organ.

[20] Cowan E, D’Ambruoso L, Van Der Merwe M, Witter S, Byass P, Ameh S, Wagner RG, Twine R (2021). Understanding non-communicable diseases: combining health surveillance with local knowledge to improve rural primary health care in South Africa. J Global Health Action.

[21] Shawon M, Hassan T, Ashrafi SAA, Azad AK, Firth SM, Chowdhury H, Mswia RG, Adair T, Riley I, AbouZahr C (2021). Routine mortality surveillance to identify the cause of death pattern for out-of-hospital adult (aged 12+ years) deaths in Bangladesh: introduction of automated verbal autopsy. J BMC public health.

[22] Ngo AD, Rao C, Hoa NP, Adair T, Chuc NTK (2010). Mortality patterns in Vietnam, 2006: Findings from a national verbal autopsy survey. J BMC research notes.

[23] Stevenson MR, Ngoan LT, Hung DV, Huong Tu NT, Mai AL, Ivers RQ, Huong HT (2012). Evaluation of the Vietnamese A6 mortality reporting system: injury as a cause of death. Inj Prev.

[24] Bui TTQ, Nguyen TTN, Pham VC. The causes of deaths in CHILILAB between 2008–2010 based on verbal autopsy method. Vietnam J Public Health. 2012;1(1):24–31.

[25] Nichols EK, Byass P, Chandramohan D, Clark SJ, Flaxman AD, Jakob R, Leitao J, Maire N, Rao C, Riley I (2018). The WHO 2016 verbal autopsy instrument: An international standard suitable for automated analysis by InterVA, InSilicoVA, and Tariff 2.0. PLoS Med.

[26] Hazard RH, Buddhika MP, Hart JD, Chowdhury HR, Firth S, Joshi R, Avelino F, Segarra A, Sarmiento DC, Azad AK (2020). Automated verbal autopsy: from research to routine use in civil registration and vital statistics systems. BMC Med.

[27] Thi Thuy Nga N, Thi My Anh B, Nguyen Ngoc N, Minh Diem D, Duy Kien V, Bich Phuong T, Quynh Anh T, Van Minh H. Capacity of commune health stations in Chi Linh district, Hai Duong Province, for prevention and control of noncommunicable diseases. J Asia Pacific Journal of Public Health. 2017;29(5):94-101. 10.1177/1010539517717020 28719767

[28] Kien VD, Van Minh H, Giang KB, Ng N, Nguyen V, Tuan LT, Eriksson M (2018). Views by health professionals on the responsiveness of commune health stations regarding non-communicable diseases in urban Hanoi, Vietnam: a qualitative study. J BMC Health Services Research.

[29] Kassa MD, Grace JM (2019). A mixed-method study of quality, availability and timeliness of non-communicable disease (NCD) related data and its link to NCD prevention: perceptions of health care workers in Ethiopia. J Health Inf Manag J.

[30] Nguyen QN, Pham ST, Nguyen VL, Wall S, Weinehall L, Bonita R, Byass P (2011). Implementing a hypertension management programme in a rural area: local approaches and experiences from Ba-Vi district Vietnam. J BMC Public Health.

[31] Long H, Ma Z, Hanh TTD, Minh HV, Rawal LB, Urmi DS, Jafar TH, Tang S, Abdullah AS (2020). Engaging village health workers in non-communicable disease (NCD) prevention and control in Vietnam: a qualitative study. Glob Public Health.

