# Supplementary material for: Putting non-communicable disease data to work in Vietnam: an investigation of community health surveillance capacity
Source: BMC Public Health. 2023 Feb 14;23:321. doi: 10.1186/s12889-023-14986-4 (PMC9926709; doi:10.1186/s12889-023-14986-4)
Supplement: Supplementary file 1 — Additional file 1: Supplementary Table 1. Data collection tools and methods. Supplementary Table 2. Planning and funding for NCD surveillance activities. Supplementary Table 3. Questionnaire sections for the self-administered survey. Supplementary Table 4. The specialized documents for NCD surveillance. Supplementary Table 5. Source of information for identifying the number of death and cause of death in CHSs. Supplementary Figure 1. Self-assessment of data quality, timeliness, and completeness. [file 12889_2023_14986_MOESM1_ESM.docx]

# SUPPLEMENTARY MATERIALS

**Supplementary Table 1.** Data collection tools and methods

| **Topic**: Implementation status of non-communicable disease (NCD) surveillance activities: mortality, morbidity and risk factor surveillance, and policy and system capacity surveillance  **Tools**: A set of questions on capacity and needs in NCD surveillance of the regional PHIs provincial Centres for Disease Control (CDC), and CHS (CHS)  **Method**: Self-completed questionnaire, in-depth interviews with leaders, and focus group discussion with staff  **Details**:   - Data collected and synthesized - Data analysis - Reporting and using information |
| --- |
| **Topic**: Factors affecting the implementation of NCD surveillance activities: policy and governance, human resources, equipment and documents, books, tools, and training materials, information technology, planning, and finances  **Tools**: A set of questions on capacity and needs in NCD surveillance within Centres for Disease Control (CDC), PHIs, and CHS  **Method**: Self-completed questionnaire  **Details**:   - Policy documents on NCD surveillance received by the PHIs/CDCs - Functions and duties of PHIs and CDCs and related units - The organisational structure of units to perform the tasks - NCD surveillance plan - Number and qualifications of staff participating in NCD surveillance in the PHIs and CDCs - Office equipment - Equipment for risk factor surveillance - Documents, instructions, surveillance forms for mortality, risk factors, statistics, reports - Current software - Online reports - Funding sources for NCD surveillance: funding for training, workshops, and implementation |
| **Topic**: Identifying capacity building needs; obstacles and solutions; training needs  **Tools**: Guide to in-depth interviewing with leaders of PHIs and CDCs and focus group discussions with CDCs, PHIs, and CHS  **Method**: In-depth interviews  **Details**:   - Obstacles in NCD surveillance - Proposed solutions for capacity building - Training contents - Forms of training - Training methods |

**Supplementary Table 2.** Planning and funding for NCD surveillance activities

|  | Public Health Institutes  (n=6) (%) | Centres for Disease Control  (n=53) (%) | Commune Health Stations  (n=148) (%) |
| --- | --- | --- | --- |
| Planning for NCD surveillance activities (either separate plan for NCD surveillance or integrated into NCD control plan) | 6 (100%) | 52 (98.1%) | 121 (81.7%) |
| Allocated funding for NCD surveillance | 5 (83.3%) | 46 (86.9%) | 39 (26.4%) |
| Funding source |  |  |  |
| Annual budget allocated for all activities | 2 (33.3%) | 8 (15.1%) | 4 (2.7%) |
| Targeted program from  the Ministry of Health | 4 (66.7%) | 27 (50.9%) | 34 (22.9%) |
| Local budget | - | 39 (73.6%) | 3 (2.0%) |
| From projects | - | 11 (20.8%) | - |

**Supplementary Table 3.** Questionnaire sections for the self-administered survey

| **Domain** | **Sub-domain with detail questions** | | | **Type of questions** |
| --- | --- | --- | --- | --- |
| **Implementation of NCD surveillance activities** | | | | |
| Mortality surveillance | - Mortality data collected and synthesized - Mortality data analysis - Reporting and using mortality information | | | Multiple/single option  Open-ended question |
| Morbidity surveillance | - Morbidity data collected and synthesized - Morbidity data analysis - Reporting and using morbidity data | | | Multiple/single option  Open-ended question |
| Surveillance of risk factors | - Risk factor data collected and synthesized - Risk factor data analysis - Reporting and using risk factor information | | | Multiple/single option  Open-ended question |
| Policy and system capacity surveillance | - Policy and system capacity data collected and synthesized - Policy and system capacity data analysis - Reporting and using policy and system capacity information | | | Multiple/single option  Open-ended question |
| **Capacity and Influencing factors** | | | |  |
| Policy and governance | | | - The policy documents on NCD surveillance received by the PHIs/CDCs - The functions and duties of the PHIs and CDCs and related units on NCD surveillance - The organizational structure of units to perform the tasks of NCD surveillance - The NCD surveillance plan | Multiple/single option  Open-ended question |
| Human resource | | | - The number and qualification of staff participating in NCD surveillance in the PHIs/ CDCs | Matrix/table  Multiple/single option  Open-ended question |
| Equipment and documents, books, tools and training materials | | | - The office equipment - The equipment for the surveillance of risk factors - The documents, instructions, surveillance forms for mortality, risk factors, statistics, reports | Multiple/single option  Open-ended question |
| Information technology | | | - The currently applied software - The online reports | Multiple/single option  Open-ended question |
| Planning and finances | | | - Funding sources for NCD surveillance: funding for training, workshop and implementation | Multiple/single option  Open-ended question |
| **Obstacles, solutions and training needs** | | | |  |
| Obstacles and solutions | | - Self-perception of NCD surveillance quality - Obstacles in NCD surveillance - Proposed solutions for capacity building | | Rating  Multiple/single option  Open-ended question |
| Training needs | | - The training contents - The forms of training - Training methods | | Rating  Multiple/single option  Open-ended question |

**Supplementary Table 4.** The specialized documents for NCD surveillance

|  | **PHI (n=6)** | | **CDC (n=53)** | |
| --- | --- | --- | --- | --- |
| Guidelines |  | |  | |
| General guidelines on NCD surveillance | 0 | | 0 | |
| Guidelines on NCD surveillance indicators | 3 (50 % ) | | 21 (39.6%) | |
| Guidelines on investigating risk factors | 6 (100%) | | 30 (56.6%) | |
| Guidelines on morbidity surveillance | 0 | | 0 | |
| Guidelines on cancer recording | 0 | | 7 (13.2%) | |
| Guidelines on mortality surveillance | 2 (33.3%) | | 9 (16.9%) | |
| Guidelines on investigation and survey of system capacity | 0 | | 0 | |
| Guidelines on statistics and reporting on NCDs | 5 (83.3%) | | 41 (77.4%) | |
| Reporting Form |  |  | |  |
| The periodic NCD statistics report form, issued by GDPM | 6 (100%) | 53 (100%) | |  |
| World Health Organization’s NCD statistical report forms | 6 (100%) | 23 (43.4%) | |  |
| Other NCD Statistical Report Forms from different Projects | 3 (50%) | 38 (71.7%) | |  |
| Methods for NCDs data statistics and reporting. |  |  | |  |
| Excel tools | - | 50 (94.3%) | |  |
| Google Drive tools | - | 32 (60.3%) | |  |
| Paper-based reports | - | 28 (52.8%) | |  |

**Supplementary Table 5.** Source of information for identifying the number of death and cause of death in CHSs and

| **Souse of Information for identifying the number of death in CHSs (n=148)** | |
| --- | --- |
| Village health workers | 105 (70.9%) |
| Population collaborators | 106 (70.9%) |
| Judicial and commune public security officials | 63 (42.6%) |
| Families and relatives | 20 (13.5%) |
| **Source of information for determining the cause of death in CHSs (n=148)** | |
| Drawing from multiple sources | 127 (85.8%) |
| Interviews with the deceased individuals’ relatives | 80 (54.1%) |
| Death certificates | 73 (49.3%) |
| Hospital discharge papers | 55 (37.2%) |
| A survey conducted with the deceased individuals’ families | 20 (13.5%) |

**Supplementary Figure 1**. Self-assessment of data quality, timeliness, and completeness

|  |  |  |
| --- | --- | --- |
